# The Education of Dentists

**Published:** 1898-07-02

**Authors:** 


### THE EDUCATION OF DENTISTS.

Growls are beginning to be heard about the curriculum for the dental diploma and the character of the examinations on the faith of which the colleges are passing men as qualified dentists. There is much reason to fear that the time now spent by many dental students in getting up the smattering of medical knowledge required of them might be far better employed in attaining that practical skill in dealing with disorders and deficiencies of the teeth, the possession of which, indeed, is the sole excuse for the existence of dentistry as a profession apart from general medicine and surgery. A well-qualified surgeon ought certainly to know about teeth just as he knows about eyes, ears, or club feet, and if, as is true enough, specialists ought to know still more, specialists can be had from within the ranks of the profession. Manual skill in dealing with dental disorders is, however, another matter. Scaling, extracting, stopping, boring little holes here, there, and everywhere, and making all sorts of curious mechanical contrivances for restoring beauty and masticating power, must always remain the special work of a special set of men. This is an art which is quite distinct from medicine, surgery, and obstetrics; it is an art so exacting as to be sufficient for a man's life-work, and no one will deny that the public, in trusting their teeth to the care of those who call themselves dentists, ought to have some security that such people are thoroughly qualified to perform these manipulations. Hence the examination and registration of dentists. The question is, does the prescribed curriculum and do the prescribed examinations as at present conducted, tend to secure for the use of the public and the medical profession a class of men who are thoroughly competent in these more or less mechanical processes, and thoroughly skilful in dentistry pure and simple; and do they not rather tend to turn out a set of men who, however wide may be their general in regard to medical and anatomical affairs, are less to be trusted for the real work they have to do than they would be had they, even at the expense of a less wide education, gone through a longer term of apprenticeship to the actual business of their lives.

